# Acute hypoxic respiratory failure due to Lenalidomide-induced interstitial pneumonitis in a patient with multiple myeloma

**DOI:** 10.1186/s12890-024-03129-z

**Published:** 2024-07-04

**Authors:** Kyle T. O’Meara, Kush Fansiwala, Nikhita Kathuria-Prakash, Monica El-Masry, Scott Oh

**Affiliations:** 1 Cedars Sinai Medical Center, Los Angeles, USA; 2 University of California, Los Angeles, Los Angeles, USA; 3 University of Southern California, Los Angeles, USA

**Keywords:** Pneumonitis, Lenalidomide, Hypoxia, Respiratory failure, Multiple myeloma

## Abstract

**Background:**

Patients with multiple myeloma are immunosuppressed due to both the disease itself and immunosuppressive therapies. Thus, when presenting with respiratory failure and pulmonary opacities, pneumonia must be considered. However, while rare, immunomodulating medications used in the treatment of multiple myeloma can also cause potentially life-threatening respiratory failure, a distinction which has important treatment implications.

**Case presentation:**

An 80-year-old male with recently diagnosed multiple myeloma undergoing treatment with lenalidomide and daratumumab presented with acute, rapidly progressive hypoxic respiratory failure ultimately requiring intubation and mechanical ventilatory support. Imaging revealed bilateral pulmonary opacities, however infectious workup was negative, and he was ultimately diagnosed with lenalidomide-induced interstitial pneumonitis, a rare but serious adverse effect of this medication. He was treated with drug discontinuation and methylprednisolone, and quickly recovered.

**Conclusion:**

Lenalidomide is an immunomodulating medication used in the treatment of multiple myeloma, and is associated with rare but serious cases of drug-induced interstitial pneumonitis. Thus, if a patient receiving lenalidomide develops shortness of breath and/or hypoxia, drug-induced pneumonitis must be on the differential. Permanent drug discontinuation with or without corticosteroids is the mainstay of treatment, and patients are often able to fully recover, underscoring the need for early recognition of this condition.

## Background

Patients with multiple myeloma are functionally immunosuppressed as a result of both the hematologic malignancy itself and the use of immunosuppressive drugs for treatment. Multiple myeloma is typically treated with a cocktail of medications, including immunomodulatory agents and steroids, followed by high doses of chemotherapy and autologous stem cell rescue in transplant eligible patients. Concomitant administration of multiple immunosuppressive medications increases the risk of opportunistic infection, and in patients with myeloma presenting with respiratory failure and pulmonary opacities, antimicrobial therapy targeting pulmonary pathogens is generally warranted. However, while rare, the immunomodulating agent lenalidomide used in the treatment of multiple myeloma has been associated with severe drug-induced interstitial pneumonitis, which may clinically be confused for pneumonia. Lenalidomide-induced pneumonitis responds rapidly to drug discontinuation and corticosteroids, and patients often completely recover, underscoring the need for early recognition and treatment.

## Case presentation

An 80-year-old man presented to the emergency department for worsening dyspnea and dry cough over four days with associated fever, fatigue and non-exertional chest tightness. Past history was notable for gastroesophageal reflux disease, supraventricular tachycardia, and multiple myeloma.

He denied orthopnea, paroxysmal nocturnal dyspnea, and lower extremity swelling. The dyspnea was mild; he was still able to carry out his activities of daily living without limitation. The patient lives in Southern California and denied any recent travel or sick contacts, although he recently had a large family gathering just prior to the onset of symptoms. A prior endoscopic evaluation had noted Barrett’s esophagus, however repeat surveillance scans demonstrated resolution. He denied odynophagia or dysphagia. His review of systems was otherwise unrevealing. He had a 20 pack-year smoking history and had quit approximately 25 years prior. He denied recent alcohol or other recreational drug use. He had no known personal or family history of pulmonary disease or heart failure.

The patient previously had macrocytosis without anemia for over ten years, which was felt to be related to consumption of one to two glasses of wine daily. Eventually he developed mild anemia and his primary care provider ordered a serum protein electrophoresis, which revealed an IgG Kappa M spike of 3.37 g/dL, consistent with IgG kappa multiple myeloma. Bone marrow biopsy and aspiration showed plasma cell myeloma comprising 70% of marrow cellularity, staining positive for CD56, CD138, and kappa. In-situ hybridization studies showed trisomy 5, 13q deletion, and 5’ IGH deletion.

Approximately eight weeks prior to admission, he started treatment with daratumumab, lenalidomide (initially 25 mg daily, then reduced to 20 mg on days 1–21 of a 28-day cycle), and dexamethasone (20 mg weekly). Prophylactic acyclovir and aspirin were initiated at the start of treatment. He tolerated the initial cycle of the chemotherapy well, apart from dysphonia thought to be related to lenalidomide, and his dose was decreased to 20 mg daily for his second cycle. His last dose of daratumumab (cycle two, day eight) was completed six days prior to presentation, while lenalidomide and dexamethasone were continued up to the day of admission (cycle two, day fourteen). His only other outpatient medications were diltiazem and omeprazole.

Initial vitals were notable for a low-grade fever to 37.9 C, mild tachypnea with a respiratory rate in the 20s, and hypoxia to 89% while breathing ambient air which corrected to 95% on 2 L of oxygen per minute via nasal cannula. Heart rate and blood pressure were normal. His physical exam revealed normal heart sounds, rhonchi in all lung fields bilaterally, and no accessory muscle use. Estimated jugular venous pressure was normal and there was no lower extremity edema. Overall, he was well appearing, without conversational dyspnea.

**Fig. 1:**
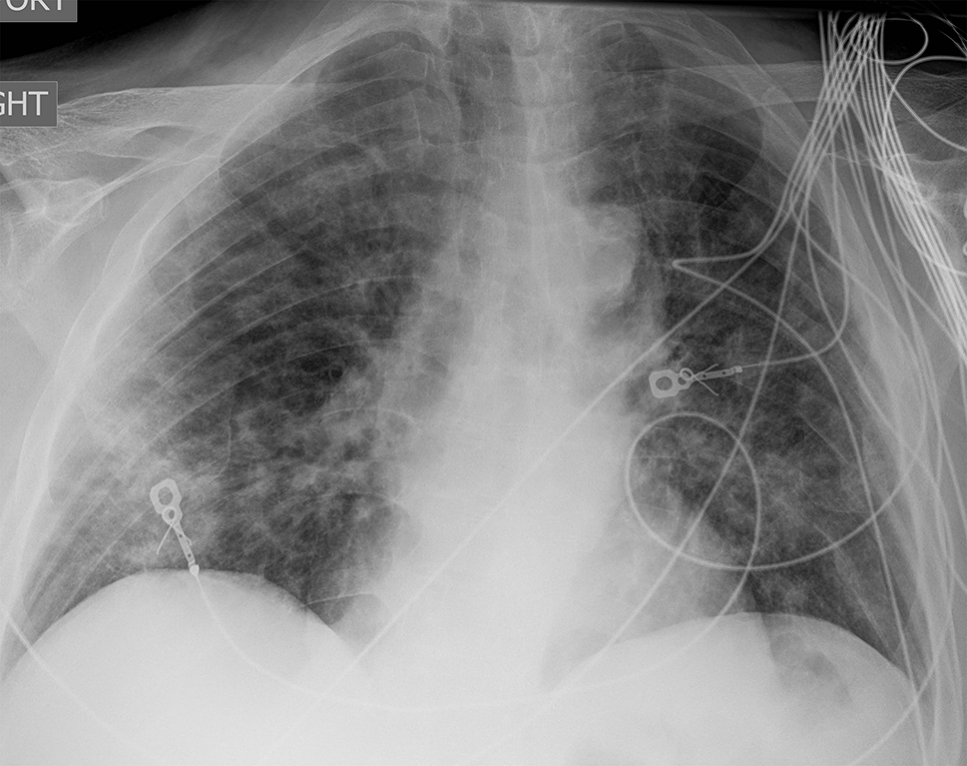
Chest X-Ray on admission demonstrating bilateral heterogeneous pulmonary opacities

A complete blood count and differential, basic metabolic panel, and liver chemistries were all within normal limits. Procalcitonin was mildly elevated at 0.3 µg/L, and lactate was normal. Brain natriuretic peptide was increased to 151 pg/mL. electrocardiogram demonstrated normal sinus rhythm, and no deep vein thrombosis was identified on ultrasound. Chest radiograph demonstrated bilateral heterogenous opacities without significant effusions (Fig. 1). Initial viral workup including COVID-19 and influenza testing was negative, and the patient was started on ceftriaxone and azithromycin for presumed community acquired pneumonia.

**Fig. 2:**
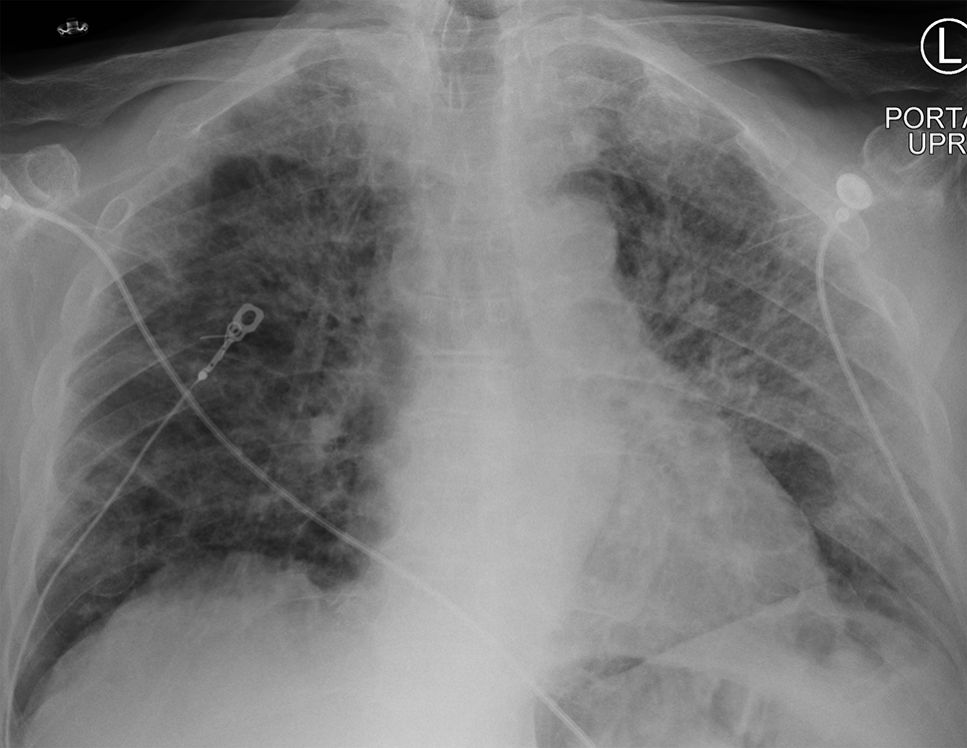
Interval Chest X-Ray approximately 10 h after admission, re-demonstrating bilateral but with significantly worsening left sided pulmonary opacities

Within approximately 10 h of admission, the patient developed rapidly progressive hypoxia with unchanged tachypnea in the twenties. An arterial blood gas drawn on 10 L of oxygen via non-rebreather mask revealed a pH of 7.47, partial pressure of carbon dioxide at 29, and a partial pressure of oxygen of 56. Repeat chest radiograph demonstrated progression of the bilateral airspace opacities to a predominantly left-sided consolidation (Fig. 2). Antibiotics were broadened to vancomycin and piperacillin-tazobactam, and the patient was transferred to the intensive care unit. Oxygen requirements continued to increase, and the patient ultimately required intubation and mechanical ventilatory support for acute hypoxic respiratory failure.

**Fig. 3:**
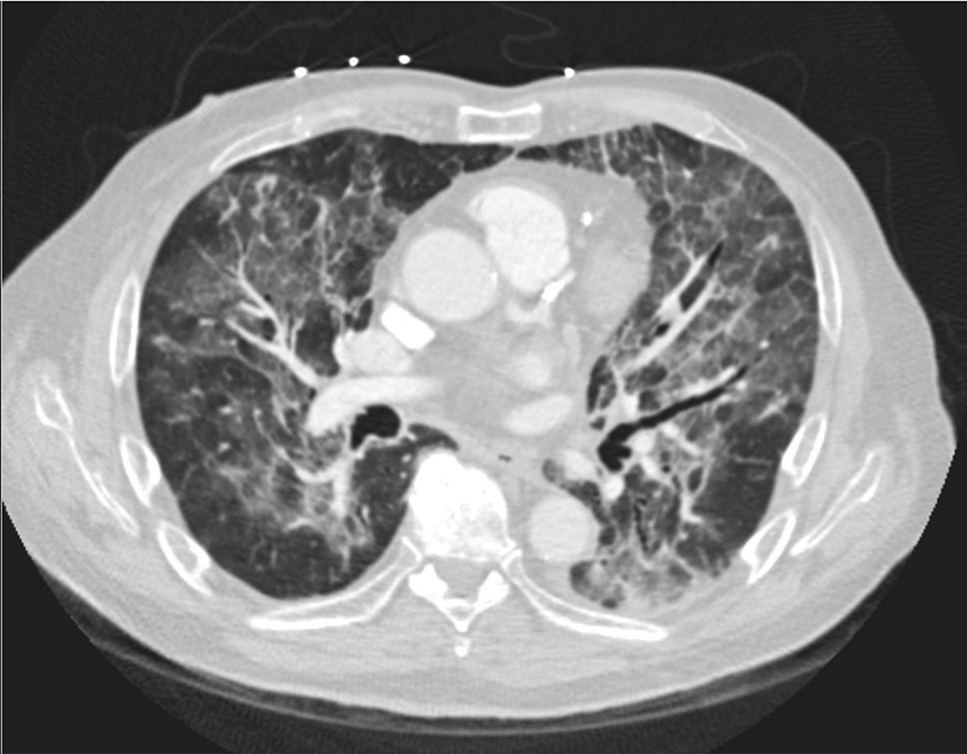
Computerized Tomography (CT) scan demonstrating bilateral ground glass opacities with concurrent diffuse consolidative and nodular opacities

Computed tomography (CT) of the chest demonstrated bilateral ground glass opacities with concurrent diffuse consolidative and nodular opacities (Fig. 3), as well as bilateral segmental and subsegmental pulmonary emboli. Of note, prior cross-sectional imaging of the chest had only demonstrated a small 3 mm pulmonary nodule, no evidence of consolidation or other opacities.

At this stage, given the imaging findings and underlying immunocompromise, there remained concern for an infectious etiology, including viral, bacterial or fungal pathogens. However, the differential diagnosis also included drug-induced pneumonitis, as this is a previously reported adverse effect of lenalidomide.

Following intubation, patient underwent bronchoscopy which demonstrated unremarkable airways. Bronchoalveolar lavage (BAL) was performed with cytology negative for malignancy and without eosinophilia. BAL microbiologic studies were negative including for fungi (including pneumocystis jirovecii, cryptococcus and coccidiomycosis), bacteria (including mycoplasma, mycobacterium, chlamydia, and legionella), and viral pathogens (including cytomegalovirus, parainfluenza, metapneumovirus, and respiratory syncytial virus).

Methylprednisolone (50 mg daily for 7 days) was initiated for presumed drug-induced interstitial pneumonitis in the setting of lenalidomide treatment, along with therapeutic anticoagulation for pulmonary emboli, with rapid improvement in gas exchange. The patient was successfully liberated from the ventilator within several days, and quickly weaned to minimal supplemental oxygen needs via simple nasal cannula at 2 L of oxygen per minute. He was discharged to complete a seven-week prednisone taper. Lenalidomide had been held since admission and was discontinued at discharge.

## Discussion and conclusions

We present a case of an 80-year-old male with multiple myeloma undergoing treatment with daratumumab, lenalidomide and dexamethasone, who developed rapidly progressive acute hypoxic respiratory failure with bilateral ground glass pulmonary opacities complicated by profound hypoxic respiratory failure requiring mechanical ventilatory support. Both daratumumab and lenalidomide are associated with an immunosuppressive effect and an increased risk of infections, including pneumonia [1, 2]. However, the patient deteriorated while on broad-spectrum antibiotics, and extensive microbiologic investigations were unremarkable. This prompted the use of systemic steroids for drug-induced interstitial pneumonitis, resulting in rapid improvement. Importantly, lenalidomide is the only prior to admission medication for our patient that is associated with interstitial lung disease; daratumumab is not known to cause significant pulmonary toxicity.

Lenalidomide is an immunomodulating thalidomide analog. It is widely used as first line treatment for multiple myeloma given a favorable side effect profile, improvement in progression-free survival, and an overall survival benefit compared to the prior standard therapy (melphalan, prednisone, and thalidomide or bortezomib) in elderly patients who are ineligible for autologous stem cell transplant [3]. In 2016, it was approved by the Food and Drug Administration as a first-line therapy for patients with multiple myeloma [4]. While the mechanism of action is not fully understood, it has immunomodulatory, antiangiogenic, and antineoplastic effects, inhibits proliferation and induces apoptosis of multiple myeloma tumor cells [5]. Pneumonitis is currently listed as a potential severe reaction of lenalidomide, but is a relatively rare adverse effect.

Lenalidomide-induced pneumonitis has previously been described [6–11]. Presenting symptoms include dry cough, dyspnea, fever, and fatigue. Nearly all previous cases involve patients taking only dexamethasone and lenalidomide, with no other concurrent immunomodulatory agents. In several cases, symptoms started within several weeks of initiating treatment, similar to our patient. However, more delayed reactions have also been reported [9]. While most cases describe moderately severe disease characterized by hypoxia requiring hospitalization [6–8], a more subacute presentation managed in the outpatient setting has also been described [11]. There are even fewer reports of fulminant courses complicated by profound hypoxia requiring mechanical ventilatory support, such as the one seen in our case [9, 10].

Similar to our patient, the patients in these reports had unremarkable blood counts and negative infectious workups, including on bronchoalveolar lavage and biopsies. Reported chest radiography findings have been inconsistent; while in some cases they have been unremarkable [6, 11], new prominent interstitial markings or alveolar opacities have been found in the others [7–9]. Our patient notably did have bilateral heterogenous opacities on chest X-ray with brisk evolution. In contrast with chest radiography, however, CT chest has consistently demonstrated extensive ground glass opacities, and would seem to be the imaging modality of choice. Transbronchial biopsies in previous cases have demonstrated non-specific inflammation, organizing pneumonia, or lymphocytic infiltration [6–8].

In agreement with previously described cases of lenalidomide-induced interstitial pneumonitis complicated by acute hypoxic respiratory failure, our patient was treated with drug discontinuation and corticosteroids [7, 9, 10]. His respiratory symptoms and oxygenation promptly improved over the course of several days and returned to baseline over several weeks. While in some reports drug discontinuation alone has resulted in clinical and radiographic improvement [6, 11], in at least one case it was insufficient and subsequent initiation of steroids was required [8]. Thus, the question remains as to whom can be safely treated with drug discontinuation alone. The decision to utilize steroids was made in our case because the patient was critically ill with severe respiratory failure, with the hope that aggressive anti-inflammatory treatment would facilitate faster recovery than drug discontinuation alone.

Importantly, in one report, when the patient was rechallenged with lenalidomide, symptoms recurred [6]. Accordingly, while this particular adverse effect is rare, it necessitates transition to alternative therapies; per the manufacturers, permanent drug discontinuation is the primary management strategy of lenalidomide-induced pneumonitis [5]. Of note, rare cases of pulmonary toxicity manifesting as interstitial pneumonitis have also been reported with the other immunomodulatory agents used in the treatment of multiple myeloma, thalidomide and pomalidomide, although none severe enough to require mechanical ventilation [12–14].

Although our patient was found to have bilateral segmental and subsegmental pulmonary emboli, the overall clinical course, degree of hypoxic respiratory failure, and imaging findings were not consistent with pulmonary emboli being the lone etiology for respiratory failure. Malignancy, including multiple myeloma, is a risk factor for venous thromboembolism [15], and lenalidomide treatment further increases this risk, particularly when co-administered with dexamethasone [2, 16]. Up to 5% of patients undergoing treatment with lenalidomide and dexamethasone will develop deep vein thrombosis, and 4% will develop pulmonary embolism, despite thromboprophylaxis with either low dose aspirin, low molecular weight heparin, heparin, or warfarin [17].

In conclusion, this case report emphasizes that if a patient receiving lenalidomide develops shortness of breath and/or hypoxia, lenalidomide-induced interstitial pneumonitis must be on the differential. Permanent drug discontinuation with or without corticosteroids are the mainstay of treatment, and patients are often able to fully recover.

## Data Availability

Not applicable.

## Abbreviations

CT: Computerized tomography
BAL: Bronchoalveolar lavage
